# Knowledge of cardiovascular risk factors among caretakers of outpatients attending a tertiary cardiovascular center in Tanzania: a cross-sectional survey

**DOI:** 10.1186/s12872-020-01648-1

**Published:** 2020-08-10

**Authors:** Pedro Pallangyo, Nsajigwa Misidai, Makrina Komba, Zabella Mkojera, Happiness J. Swai, Naairah R. Hemed, Henry Mayala, Smita Bhalia, Jalack Millinga, Upendo W. Mollel, Happiness L. Kusima, Ester Chavala, Ziada Joram, Halifa Abdallah, Rajabu Hamisi, Mohamed Janabi

**Affiliations:** 1 Unit of Research and Training, Jakaya Kikwete Cardiac Institute, P. O Box 65141, Dar es Salaam, Tanzania; 2 Department of Adult Cardiology, Jakaya Kikwete Cardiac Institute, P. O Box 65141, Dar es Salaam, Tanzania; 3 Department of Nursing, Jakaya Kikwete Cardiac Institute, P. O Box 65141, Dar es Salaam, Tanzania; 4 Outpatient Department, Jakaya Kikwete Cardiac Institute, P. O Box 65141, Dar es Salaam, Tanzania; 5 Quality Assurance Unit, Jakaya Kikwete Cardiac Institute, P. O Box 65141, Dar es Salaam, Tanzania

**Keywords:** Cardiovascular disease knowledge, Health literacy, Health knowledge, Awareness, CVD risk knowledge, Tanzania

## Abstract

**Background:**

Health literacy on cardiovascular diseases (CVDs) plays an effective role in preventing or delaying the disease onset as well as in impacting the efficacy of their management. In view of the projected low health literacy in Tanzania, we conducted this cross-sectional survey to assess for CVD risk knowledge and its associated factors among patient escorts.

**Methods:**

A total of 1063 caretakers were consecutively enrolled in this cross-sectional study. An adopted questionnaire consisting of 22 statements assessing various CVD risk behaviors was utilized for assessment of knowledge. Logistic regression analyses were performed to assess for factors associated with poor knowledge of CVD risks.

**Results:**

The mean age was 40.5 years and women predominated (55.7%). Over two-thirds had a body mass index (BMI) ≥25 kg/m^2^, 18.5% were alcohol drinkers, 3.2% were current smokers, and 47% were physically inactive. The mean score was 78.2 and 80.0% had good knowledge of CVD risks. About 16.3% believed CVDs are diseases of affluence, 17.4% thought CVDs are not preventable, and 56.7% had a perception that CVDs are curable. Low education (OR 2.6, 95%CI 1.9–3.7, *p* < 0.001), lack of health insurance (OR 1.5, 95%CI 1.1–2.3, *p* = 0.03), and negative family history of CVD death (OR 2.2, 95%CI 1.4–3.5, p < 0.001), were independently associated with poor CVD knowledge.

**Conclusions:**

In conclusion, despite of a good level of CVD knowledge established in this study, a disparity between individual’s knowledge and self-care practices is apparent.

## Background

As the pervasive struggle against infectious diseases continue, sub Saharan Africa (SSA) is facing a rapid epidemiological transition characterized by an increasing predominance of chronic diseases particularly those affecting the cardiovascular system [1]. Although the ever-present communicable diseases remain the leading contributors to disease burden in the SSA region, non-communicable diseases (NCDs) are escalating at an alarming pace and it is projected that coming 2030 they will become the leading cause of morbidity and mortality [2, 3]. Nevertheless, low-middle income countries (SSA region inclusive) are currently witnessing a disturbingly disproportionate share of global NCDs deaths (i.e. > 75%) [4]. Owing to urbanization and sedentary life-style adoption, several NCD risk factors (i.e. smoking, heavy drinking, unhealthy diets, physical inactivity and overweight) are increasingly widespread in SSA communities and are postulated to be the drivers of the rapidly growing CVD burden in the region [4–6].

By virtue of their chronic nature, CVDs are of long duration and generally slow in progression necessitating life-long care inevitably with continuous expenditure [3, 5]. Nonetheless, these conditions are largely preventable through life-style modification to curb the exposure to the established risk factors [1–3]. It is evident that health literacy of CVD risk factors plays a considerably effective role in preventing or delaying the onset of disease as well as in impacting the efficacy of their management [7–10]. Likewise, persons with low functional health literacy have been associated with diminished use of the health system, less likelihood of engaging in health-promoting behaviors and poorer overall health outcomes [7–10].

Whilst SSA region is having one of the lowest adult literacy rates (65%) [11] in the world, just about a third of the Tanzanian population is estimated to have adequate health literacy [12]. Several studies have addressed the growing burden and pattern of CVD risk factors; however, there is dearth of information regarding public knowledge of CVD risk factors in SSA region particularly Tanzania. In this cross-sectional survey, we sought to assess the CVD risk knowledge and its associated factors among companions of outpatients attending a tertiary-level cardiovascular hospital in Tanzania. Such estimation of the baseline knowledge regarding CVD risks has potential public health relevance particularly in the development of targeted educational programs which are pivotal amidst the rapidly rising crisis.

## Methods

### Recruitment process and definition of terms

A cross-sectional survey was conducted between December 2019 and February 2020 at Jakaya Kikwete Cardiac Institute (a tertiary care public teaching hospital) in Dar es Salaam, Tanzania. A consecutive sampling method was utilized to recruit consented individuals who escorted known patients with CVD for a scheduled clinic visit. A structured questionnaire bearing questions pertaining to sociodemographic and clinical characteristics, measurement of key vitals (blood pressure, blood sugar, height, weight and waist circumference), and standard questions for assessing CVD risk knowledge was utilized. Physical activity was assessed using the Physical Activity Vital Sign (PAVS) scale [13]; with scores of 0 min/week denoting inactivity, 1 - < 150 min/week signifying underactivity and ≥ 150 min/week indicating physical activeness. We defined underweight as BMI < 18.5 kg/m^2^, normal: BMI 18.5–24.9 kg/m^2^, overweight: BMI 25.0–29.9 kg/m^2^ and obese: BMI ≥30.0 kg/m^2^ [14]. Individuals who smoked at least 1 cigarette in the past 6 months were regarded as current smokers, those who last smoked over 6 months or self-reported quitting smoking were considered past smokers and those who never smoked were regarded as non-smokers. Alcohol drinking was defined as at least a once consumption every week. Hypertension was defined as SBP ≥140 mmHg or DBP ≥90 mmHg, or use of blood pressure lowering agents [15]. Diabetes was diagnosed using a random blood glucose (RBG) ≥11.1 mmol/L and/or fasting blood glucose (FBG) ≥7 mmol/L or use of glucose-lowering agents [16]. An adopted questionnaire consisting of 22 statements assessing various CVD risk behaviors was utilized for assessment of knowledge [17]. A percentage score for each participant was computed by dividing the sum of correct responses divided by the total number of questions (i.e. 22) multiplied by 100. A score of < 50% was classified as low; 50–69% moderate and ≥ 70% good knowledge [18, 19].

### Statistical analysis

All statistical analyses were performed by STATA v11.0 software. Summaries of continuous variables are presented as means (± SD) and categorical variables are presented as frequencies (percentages). Categorical and continuous variables were compared using the Pearson Chi square test and student’s T-test respectively. Bivariate analyses were performed to assess for factors associated with poor knowledge of CVD risks. Factors included in this analysis were age, sex, education level, marital status, employment status, residence, possession of health insurance, BMI, self-perceived health status, medical check-up history, self-reported knowledge of CVDs, family history of CVD, family history of CVD-related death, physical activity, smoking status, alcohol use, dietary habits, hypertension and diabetes history. Wald Chi-Square tests was used to assess for the interaction terms, with a *p* < 0.05 cut-off used as criteria for inclusion in multivariate analysis. Variables maintained in the multivariate model underwent stepwise and backward selection procedures. Odd ratios with 95% confidence intervals and *p*-values are reported. Statistical significance was set at p < 0.05 and all tests were two tailed.

## Results

### Study population

A total of 1063 individuals who escorted outpatients with established diagnosis of CVD were consecutively enrolled in this study. Table 1 displays the sociodemographic and clinical characteristics of the study participants. Their mean age was 40.5 years and there was a female predominance (55.7%). Majority (59%) of participants had at least secondary school education and 79.4% had a regular income generating activity. Over 85% of participants resided in urban areas and just over a third were health insured. Regarding participants’ relationship to the patient: 13.4% were spouses, 62.2% were children, 15.2% were siblings, 3.6% were parents and 5.6% were friends. Over two-thirds (66.8%) of participants had a BMI ≥ 25, 18.5% were alcohol drinkers, 3.2% were current smokers, 17.8% reported a regular healthy eating, and 47% were physically inactive. Nearly one-fifth (19.2%) of participants had a personal history of hypertension and 4.1% were known to have diabetes mellitus.

**Table 1:** Characteristics of participants in survey assessing knowledge of CVD risk factors (*N* = 1063)

Characteristic	Proportion (%)
**Age** (Mean, SD)	40.5 (13.0)
Range	18–77
**Age group**	
18–34	379 (35.7%)
35–54	525 (49.3%)
≥ 55	159 (15.0%)
**Sex**	
Male	471 (44.3%)
Female	592 (55.7%)
**Education**	
No Formal	22 (02.1%)
Primary	404 (38.0%)
Secondary	385 (36.2%)
University	252 (23.7%)
**Marital status**	
Single	279 (26.3%)
Married	711 (66.9%)
Divorced	26 (02.5%)
Widowed	47 (04.4%)
**Occupation**	
Jobless	112 (10.5%)
Student	54 (05.1%)
Self-employed	640 (60.2%)
Employed	204 (19.2%)
Retired	53 (05.0%)
**Residence**	
Urban	907 (85.3%)
Rural	156 (14.7%)
**Region of Residence**	
Dar es Salaam	752 (70.7%)
Other regions	311 (29.3%)
**Relationship to Patient**	
Spouse	142 (13.4%)
Child	661 (62.2%)
Sibling	162 (15.2%)
Parent	38 (03.6%)
Friend	60 (05.6%)
**Health insured**	
Yes	358 (33.7%)
No	705 (66.3%)
**Perceived health status**	
Good	439 (41.3%)
Average	577 (54.3%)
Bad	47 (04.4%)
**When last check-up**	
Never	583 (54.9%)
Within a Year	399 (37.5%)
Over a Year	81 (07.6%)
**Personal Disease History** (% Yes)	
CVD/Hypertension	204 (19.2%)
Diabetes	43 (04.1%)
Chronic kidney disease	12 (01.1%)
HIV/AIDS	26 (02.5%)
Cancer	6 (0.6%)
Chronic pulmonary disease	19 (01.8%)
Chronic back pain	83 (07.8%)
**Knowledge of CVD risk factors** (self-reported)	
Yes	366 (34.4%)
No	697 (65.6%)
**Family history of CVD**	
Yes	389 (36.6%)
No	661 (62.2%)
Don’t know	13 (01.2%)
**CVD death in the family**	
Yes	218 (20.5%)
No	799 (75.2%)
Don’t know	46 (04.3%)
**Smoking status**	
Current	34 (03.2%)
Past	43 (04.0%)
Never	986 (92.8%)
**Alcohol intake**	
Yes	197 (18.5%)
No	866 (81.5%)
**Perceived healthy eating**	
Irregularly	874 (82.2%)
Regularly	189 (17.8%)
**≥30 min Exercise** (days/week)	
0 days	500 (47.0%)
1–3 days	300 (28.2%)
4–6 days	80 (07.6%)
7 days	183 (17.2%)
**Body Mass Index** (mean, SD)	28.0 (5.9)
**BMI categories**	
Underweight	32 (03.0%)
Normal	321 (30.2%)
Overweight	358 (33.7%)
Obese	352 (33.1%)
**Waist circumference** (mean [cm], SD)	94.6 (13.3)
Men ≥94 cm	207 (44.0%)
Women ≥80 cm	528 (89.2%)
**Blood Pressure** (mean, SD)	
Systolic Blood Pressure	128.5 (20.2)
Diastolic Blood Pressure	83.0 (13.5)
**Blood Pressure Range**	
< 140/90	754 (70.9%)
≥ 140/90	309 (29.1%)
**Blood Sugar Range**	
FBG < 7.0/RBG < 11.1 FBG ≥ 7.0/RBG ≥ 11.1	1029 (96.8%) 34 (03.2%)

### Knowledge and attitude regarding CVD risk factors

While 583 (54.9%) of participants had never had a general health check-up before, 41.3% had a perception of being in good health while 34.4% reported to have knowledge of CVD risk factors. Table 2 summarizes responses to the 22 questions used to assess knowledge about CVD risk factors. The mean CVD knowledge score was 78.2% with a range of 31.8–100%. A total of 847 (79.7%) participants had good knowledge, 204 (19.2%) had moderate knowledge, and 12 (1.1%) had low knowledge of CVD risk factors. About 16.3% believed CVD are diseases of rich people and 42.4% were unaware that they are the leading cause of mortality globally. Additionally, 17.4% thought CVD are not preventable, 67.4% believed one may know that they have CVD based on symptoms alone and 56.7% had a perception that CVD are curable. Smoking was recognized by 77% as a CVD risk, physical inactivity by 95.6%, excessive alcohol drinking by 90.1%, overweight by 90.1%, high-salt diet by 85.9%, and elevated cholesterol by 92.9% of participants. Furthermore, while just 38.6% were aware that men have a higher risk of CVD compared to women, 65.6% acknowledged positive CVD family history as a risk, whereas 89.5 and 72.4% knew that hypertension and diabetes respectively are risk factors for CVD.

**Table 2:** Responses of the Cardiovascular Disease Knowledge Questionnaire used in this study (N = 1063)

Item	Question	Correct response	% answered correctly
Q1	Cardiovascular diseases (CVD) are diseases of rich people	No	890 (83.7%)
Q2	Smoking does not increase a risk of CVD	No	818 (77.0%)
Q3	Consuming a lot of vegetables and fruits increases the risk of CVD	No	1006 (94.4%)
Q4	Consumption of too much salt is a risk to CVD	Yes	913 (85.9%)
Q5	Having excess body weight increases ones risk of CVD	Yes	958 (90.1%)
Q6	A family history of CVD increases ones risk of acquiring CVD	Yes	697 (65.6%)
Q7	Generally, regular consumption of red meat is healthier than white meat	No	864 (81.3%)
Q8	Cardiovascular diseases are curable upon completion of described dose.	No	460 (43.3%)
Q9	Diabetes increases ones risk of CVD	Yes	770 (72.4%)
Q10	Men are at higher risk of CVDs compared to women	Yes	410 (38.6%)
Q11	Excessive alcohol drinking is dangerous to cardiovascular health	Yes	958 (90.1%)
Q12	CVDs are the leading cause of deaths globally	Yes	612 (57.6%)
Q13	A person may know that he/she has CVD based on signs and symptoms alone	No	347 (32.6%)
Q14	A person with CVD may infect a close person	No	1006 (94.4%)
Q15	High blood pressure is a risk factor of CVD	Yes	951 (89.5%)
Q16	Animal fat is healthier than plant oil	No	892 (83.9%)
Q17	Old age is a risk factor for CVD	Yes	850 (80.0%)
Q18	Stress increases ones risk of acquiring CVD	Yes	1000 (94.1%)
Q19	Exercising regularly is harmful to cardiovascular health	No	1016 (95.6%)
Q20	High cholesterol in blood prevents one from CVD	No	988 (92.9%)
Q21	CVD are not preventable	No	878 (82.6%)
Q22	Doing health check-ups frequently is harmful	No	1004 (94.5%)
**Mean score (SD), Range 78.2% (10.9), 31.8–100%**			

### Factors associated with knowledge of CVD risk factors

Table 3 displays findings of chi-square analyses of various characteristics by CVD knowledge status (i.e. score < 70% vs score ≥ 70%). Participants with low education had a higher likelihood of having poor knowledge of CVD risks compared to individuals with at least secondary education (30.8% vs 12.9%, *p* < 0.001). Moreover, individuals who possessed a health insurance displayed higher rates of good CVD knowledge compared to their uninsured counterparts (89.4% vs 75.2%, p < 0.001). Likewise, non-smokers showed a higher chance of having a good CVD knowledge compared to current smokers (80.4% vs 58.8%, *p* < 0.01). Furthermore, physically inactive participants had inferior likelihood of having good CVD knowledge compared to their physically active counterparts (77.0% vs 82.1%, *p* = 0.04). Additionally, participants with unhealthy eating pattern displayed a higher chance of having poor knowledge compared regular healthy dieters (22.3% vs 16.7%, *p* = 0.03). Participants with a positive family history of CVD death displayed a superior CVD risks knowledge compared to ones without a CVD-related death in the family, (88.5% vs 77.4%, *p* < 0.001).

**Table 3:** Bivariate analyses of potential associated factors for CVD risk knowledge (*N* = 1063)

Characteristic	n	Score < 70	Score ≥ 70	p-value
Age > 40	502	96 (19.1%)	406 (80.9%)	
Age ≤ 40	561	117 (20.9%)	444 (79.1%)	0.46
Female	592	121 (20.4%)	471 (79.6%)	
Male	471	92 (19.5%)	379 (80.5%)	0.72
≤Primary education	426	131 (30.8%)	295 (69.2%)	
≥Secondary education	637	82 (12.9%)	555 (87.1%)	**< 0.001**
Single	279	65 (23.3%)	214 (76.7%)	
Ever married	784	148 (18.9%)	636 (81.1%)	0.12
No regular income	219	52 (23.7%)	167 (76.3%)	
Regular income generating activity	844	164 (19.4%)	680 (80.6%)	0.16
Rural	156	39 (25.0%)	117 (75.0%)	
Urban	907	174 (19.2%)	733 (80.8%)	0.09
Uninsured	705	175 (24.8%)	530 (75.2%)	
Health insurance	358	38 (10.6%)	320 (89.4%)	**< 0.001**
BMI ≥ 25	710	133 (18.7%)	577 (81.3%)	
BMI < 25	353	80 (22.7%)	273 (77.3%)	0.12
Perception on self-health ≤average	624	119 (19.1%)	505 (80.9%)	
Perceive to be in good health	439	94 (21.4%)	345 (78.6%)	0.36
Never had health check-up	583	118 (20.2%)	465 (79.8%)	
Ever had a check-up	480	98 (20.4%)	382 (79.6%)	0.94
No knowledge of CVD risks (self-reported) Knowledgeable on CVD risks	697 366	171 (24.5%) 45 (12.3%)	526 (75.5%) 321 (87.7%)	**< 0.001**
No history of CVD death in family	845	191 (22.6%)	654 (77.4%)	
Positive history of CVD death	218	25 (11.5%)	193 (88.5%)	**< 0.001**
No regular exercise	500	115 (23.0%)	385 (77.0%)	
Exercises ≥1 day/week	563	101 (17.9%)	462 (82.1%)	**0.04**
irregular diet (< 5 days/week)	691	154 (22.3%)	537 (77.7%)	
Regular healthy diet (≥5 days/week)	372	62 (16.7%)	310 (83.3%)	**0.03**
Current smokers	34	14 (41.2%)	20 (58.8%)	
Non-smokers	1029	202 (19.6%)	827 (80.4%)	**< 0.01**
Current alcohol drinkers	197	40 (20.3%)	157 (79.7%)	
Non-drinkers	866	176 (20.3%)	690 (79.7%)	1.0
Positive personal history of CVD	204	35 (17.2%)	169 (82.8%)	
Negative personal history of CVD	859	181 (21.1%)	678 (78.9%)	0.21
Known to have diabetes	43	10 (23.3%)	33 (76.7%)	
Negative diabetes history	1020	206 (20.2%)	814 (79.8%)	0.62

A total of seventeen potential characteristics associated with knowledge of CVD risks were featured in logistic regression analysis, Table 4. During bivariate analyses seven out of the seventeen factors showed significant associations (i.e. *p* < 0.05) and were subsequently included in the multivariate regression model to control for confounders. At the end of multivariate regression analysis, three factors remained independently associated with poor CVD risks knowledge. These included: low education level (OR 2.6, 95%CI 1.9–3.7, p < 0.001), lack of health insurance (OR 1.5, 95%CI 1.1–2.3, p = 0.03), and negative family history of CVD death (OR 2.2, 95%CI 1.4–3.5, p < 0.001).

**Table 4:** Logistic Regression Analysis of Factors Associated with Poor knowledge of CVD risks

Age > 40	Age ≤ 40	1.2	0.9–1.6	0.2	–	–	–
Female	Male	0.9	0.6–1.2	0.3	**–**	**–**	**–**
≤Primary education	≥secondary education	2.9	2.1–3.9	**< 0.001**	2.6	1.9–3.7	**< 0.001**
Single	Ever married	0.8	0.6–1.1	0.2	**–**	**–**	**–**
No regular income activity	Employed/self-employed	1.3	0.9–1.8	0.2	–	–	–
Rural	Urban	0.8	0.5–1.2	0.3	**–**	**–**	**–**
Uninsured	Insured	2.3	1.6–3.3	**< 0.001**	1.5	1.1–2.3	**0.03**
BMI ≥ 25	BMI < 25	1.5	1.1–2.1	**< 0.01**	1.3	0.9–1.8	0.2
≤Average perception	Perceive to be good health	1.0	0.8–1.4	0.9	**–**	**–**	**–**
Never had health check-up	Positive check-up history	1.0	0.7–1.4	0.9	**–**	**–**	**–**
Irregular diet	Regular healthy diet	1.4	1.0–2.0	**0.03**	1.2	0.8–1.7	0.4
No CVD death history in family	Positive history of CVD death	2.3	1.4–3.5	**< 0.001**	2.2	1.4–3.5	**< 0.001**
Never exercise	Exercises ≥1 day/week	1.4	1.0–1.8	**0.04**	1.2	0.9–1.7	0.2
Current smokers	Non-smokers	2.9	1.4–5.8	**< 0.01**	2.1	0.9–4.6	0.08
Alcohol drinkers	Non drinkers	1.0	0.7–1.5	1.0	**–**	**–**	**–**
Positive hypertension history	Negative hypertension history	1.3	0.9–1.9	0.2	**–**	**–**	**–**
Diagnosed with diabetes	Negative diabetes history	0.8	0.4–1.7	0.6	**–**	**–**	**–**

## Discussion

As the NCD epidemic continues to accelerate amidst the ongoing infectious diseases battle, health-care systems in SSA are increasingly regarding CVDs in particular and NCDs in general as a top public health priority [20]. To curb this distressing trend, health literacy has a prominent significance in prevention of CVD both at the primary and secondary levels [7–10]. Sorensen K et al. [21] defined health literacy as the “individual’s knowledge, motivation, and competences to access, understand, appraise, and apply health information in order to make judgements and take decisions in everyday life concerning health care, disease prevention, and health promotion to maintain or improve quality of life during the life course”. Inspite of its evidence-based [7–10] benefits in NCDs prevention, variably low rates of health literacy have been documented around the globe making public health measures particularly the development and implementation of targeted educational programs challenging or ineffective.

With about four-fifths of participants having an overall adequate knowledge regarding CVD risk factors, this present study demonstrated a modest level of health literacy in an urban setting of SSA. Our rates of CVD literacy echoes findings of previous studies from South Africa [17], Iran [22] and Malaysia [23] which produced knowledge rates of 75.3, 78.7 and 81% respectively. Contrary to our findings, regional studies from Nigeria [24] (44%) and Cameroon [25] (47.5%) revealed considerably low rates of CVD literacy. This observed variability in literacy rates between cited studies could be explained by the education-level differences among study participants and diversity of tools used for knowledge assessment. With regards to knowledge of specific risk behaviors, over nine-tenth of participants in this study recognized excess body weight, physical inactivity, and excess alcohol intake as risks, while more than three-quarters acknowledged smoking, unhealthy diet, hypertension and diabetes as attributable risks.

A wide variation of knowledge rates regarding individual risk factors is observed in the literature. For instance, smoking [17, 23–33] has been recognized as a CVD risk by 36.2–93.2% of participants, excess alcohol intake by 40.7% [29]–65% [31], unhealthy diet [23–26, 28–31, 33] by 2.8–88%, physical inactivity [17, 23–31, 33] by 1.2–96%, excess body weight [23–31, 33] by 1.6–100%, hypertension [23–31, 33] by 6.2–94% and diabetes [17, 23–31] by 5.3–92.4%. Astonishingly, despite of a predominant blood-relationship between study participants and the escorted patients, just over one-third of participants realized they are living in a family with a positive CVD history and less than two-thirds were aware that it increases ones risk of CVD. In unison to our findings, studies by Awad et al.^26^ (62.6%), George et al.^27^ (68%), and Shafiq et al.^28^ (60%) revealed similar rates of recognition of family history as an attributable risk of CVD. Nonetheless, in a couple of other studies [25, 29, 30] majority (> 50%) of participants were unaware of the increased risk of acquiring CVD in the presence of a positive family history.

Irrespective of a predominant positive family history of CVD and acknowledgement of the importance of regular check-ups by large majority of participants, over a half of study subjects have never had a basic check-up their entire lives. Notwithstanding the relatively good CVD risk knowledge, risk behaviors were disproportionately high among participants of this present study. For instance, although excess body weight was recognized as a risk by over 90% of participants just one-third had a healthy weight. Similar pattern was observed with nearly 96% recognizing physical inactivity as a risk and yet just about a half of participants were physically active. Furthermore, certain risk factors (i.e. overweight, hypertension, and diabetes) revealed comparatively similar rates of knowledge to participants free from such risks. Nevertheless, current smokers, physically inactive and unhealthy eaters displayed inferior knowledge rates compared to their counterparts with healthy behaviors respectively.

## Conclusions

Despite a fairly good level of knowledge regarding CVD risk factors established in this study, a vivid disconnection between individual’s knowledge and self-care practices (i.e. CVD risk behaviors) is apparent. These findings reflects alarming public health concerns and underscore the urgent need to establish and implement wide-spread and effective educational initiatives aiming at mitigating the community’s practices towards cardiovascular risk factors.

## Data Availability

The final version of data set supporting the findings of this paper is submitted together with this manuscript to the editorial committee. All the raw data is included in this manuscript. There are no ethics restrictions preventing the sharing of the raw data.

## Abbreviations

BMI: Body mass index
CVDs: Cardiovascular diseases
DBP: Diastolic blood pressure
FBG: Fasting blood glucose
NCDs: Non-communicable diseases
RBG: Random blood glucose
SBP: Systolic blood pressure
SSA: Sub-Saharan Africa
